# Nodal signalling in *Xenopus*: the role of Xnr5 in left/right asymmetry and heart development

**DOI:** 10.1098/rsob.150187

**Published:** 2016-08-03

**Authors:** Emmanuel Tadjuidje, Matthew Kofron, Adnan Mir, Christopher Wylie, Janet Heasman, Sang-Wook Cha

**Affiliations:** 1 Department of Biological Sciences, Alabama State University, 1627 Hall Street, Montgomery, AL 36101, USA; 2 Cincinnati Children's Research Foundation, 3333 Burnet Avenue, Cincinnati, OH 45229, USA

**Keywords:** Xnr5, Xnr1, laterality, organogenesis, nodal

## Abstract

Nodal class TGF-β signalling molecules play essential roles in establishing the vertebrate body plan. In all vertebrates, nodal family members have specific waves of expression required for tissue specification and axis formation. In *Xenopus laevis*, six nodal genes are expressed before gastrulation, raising the question of whether they have specific roles or act redundantly with each other. Here, we examine the role of Xnr5. We find it acts at the late blastula stage as a mesoderm inducer and repressor of ectodermal gene expression, a role it shares with Vg1. However, unlike Vg1, Xnr5 depletion reduces the expression of the nodal family member *xnr1* at the gastrula stage. It is also required for left/right laterality by controlling the expression of the laterality genes *xnr1, antivin* (*lefty*) and *pitx2* at the tailbud stage. In Xnr5-depleted embryos, the heart field is established normally, but symmetrical reduction in Xnr5 levels causes a severely stunted midline heart, first evidenced by a reduction in *cardiac troponin* mRNA levels, while left-sided reduction leads to randomization of the left/right axis. This work identifies Xnr5 as the earliest step in the signalling pathway establishing normal heart laterality in *Xenopus*.

## 1. Introduction

Nodal class TGF-β signalling molecules play essential roles in establishing the vertebrate body plan. In mice, the proximal–distal and then the anterior–posterior axes of the egg cylinder are demarcated by specifically localized nodal transcripts [1]. Secreted nodal protein is believed to initiate signalling gradients across these axes [2]. The earliest dorsal–ventral asymmetry in zebrafish is the dorsal localization of *squint* mRNA at the 4–8 cell stage [3], while in *Xenopus*, all six nodal-related mRNAs (Xnr1–6) are dorsally enriched at the blastula stage [4–8]. As development proceeds in chicks and mice, new waves of nodal expression occur asymmetrically in the left/right axis of the organizer and lateral plate mesoderm (LPM) [9–12], while in zebrafish and *Xenopus*, *cyclops*, *southpaw* and *xnr1* (the same as *nodal homolog 1*), mRNAs are enriched in the left LPM [12–16]. Although many aspects of the functions of nodal type TGF-βs are known in these locations, many questions remain, particularly the individual roles of Xnrs in *Xenopus*. Previous attempts at answering this question has led to the conclusion that Xnr5 (the same as *nodal homolog 5*) and Xnr6 (the same as *nodal homology 6*) function redundantly to induce mesoderm, while Xnr1 and Xnr2 (the same as *nodal homolog 2*) redundantly control gastrulation later on [17]; however, there is no previous report of a function of Xnr5 beyond gastrulation. We re-examined the function of Xnr5 using two independent and easily verifiable loss-of-function strategies.

The *xnr5* gene is regulated by maternal transcription factors, including *vegt*, *tcf3*, *foxH1* and *Sox3* [8,18–21]. It is expressed only during a narrow time window at the blastula to gastrula stage [8,19,22]. We demonstrate that at the gastrula stage, Xnr5 acts additively with the maternally expressed TGF-β, Vg1 (the same as *gdf1*), causing Smad2 phosphorylation, Erk (the same as *mapk1*) phosphorylation, mesoderm induction and suppression of ectodermal gene (foxi1E) expression. However, unlike Vg1, Xnr5 depletion reduces the expression of the nodal family member *xnr1* at the gastrula stage. It is also essential for the correct level of expression of laterality genes at the tailbud stage including *xnr1, antivin* (the same as *lefty*) and *pitx2* mRNA. We confirmed this phenotype by CRISPR/Cas as an alternative loss-of-function approach. Deletion mutations leading to the truncation of Xnr5 protein caused the mutant embryos to lose the expression of *xnr1* in the left LPM at the tailbud stage, while the hindbrain/LPM marker *meis3* was not affected. Furthermore, we show that Xnr5-depleted embryos establish the heart field, but that reduction of Xnr5 on both sides of the embryo causes a severely stunted, midline heart, and reduced expression of the cardiac specific marker, *cardiac troponin* (the same as *tnni3*). By contrast, reduction of Xnr5 only on the left-hand side leads to randomization of the left/right axis. This work identifies Xnr5 as the earliest zygotic factor in the signalling pathway establishing normal heart laterality and morphogenesis.

## 2. Material and methods

### 2.1. Embryos and oocytes production and handling

Embryos and oocytes production, handling and injection were as previously described [23].

### 2.2. Morpholino oligonucleotide design and injection

Intron boundaries were predicted by aligning the *xnr5* cDNA to the *X. tropicalis* genomic sequence. Primers (U: 5′-GGAAAAAGACTCCCCCATGA-3′; D: 5′-ATCCACTTGGGTGCATCCT-3′) were designed to span intron 1, PCR was performed using *X. laevis* genomic DNA as template and the product was sequenced. Two pseudo-alleles were identified, and a splice donor blocking morpholino oligonucleotide (MO) (Xnr5-SD-Mo: 5′-TAAGTTACCTTTGCAATGAGGCTC-3′) was designed to target both of them. Intron-spanning primers were used to detect the spliced and unspliced forms of *xnr5* mRNA by gel-based PCR. In injection experiments, the working concentration of Xnr5 MO was a total of 60 ng per embryo, injected into the vegetal areas of all four cells at the four-cell stage, unless otherwise noted in the text. A mismatch morpholino (Xnr5MMMO: 5′-TAACTTAGCTTTTCCAATCAGGTC) was used as the control. Vg1 phosphorothioate-phosphodiester oligo (5 ng per oocyte) was used as described previously [24]. For Vg1/Xnr5 depletion experiments, the oligos were injected into oocytes rather than fertilized eggs, and fertilized by the host-transfer procedure [24].

### 2.3. Production of guide RNA and evaluation of gene targeting efficiency in Cas9/guide RNA-injected embryos

The nuclear localization signal tagged *Streptococcus pyrogenes* Cas9 protein was used according to manufacturer's instruction (PNA Bio). To create guide RNA (gRNA), we used direct jointing method with forward primer that has the target sequence and reverse primer that has the tracRNA backbone. The resulting PCR product was transcribed using the T7 Megashortscript kit (Ambion) and purified with acidic phenol/isopropanol precipitation. *Xenopus laevis* embryos were injected at one-cell stage with a mixture of 600 pg NLS-SpCas9 protein and 300 pg of each Xnr5 gRNA, and cultured at 23°C until they reached the desired stage. When injected embryos reached stage 20, we randomly pooled five healthy embryos from each injection, extracted total RNA and performed real-time PCR (RT-PCR) to amplify the targeted region of *xnr5* genes by PCR (for primers, see electronic supplementary material, table S3), and then cloned the purified PCR products into the pCRII-TOPO vector (Invitrogen). Twenty single colonies were randomly picked for DNA sequencing analysis to detect any insertion or deletion (indel) mutations resulting from error-prone non-homologous end joining (NHEJ)-based repair of Cas9-created double-strand breaks. The gRNA targeting efficiency was determined by the ratio of mutant to total colonies.

### 2.4. Analysis of potential off-target effects of the guide RNA

All genome loci containing up to four mismatches compared with the gRNA's 20-nucleotide target sequence were identified by GGGenome ultrafast search method. For each injection, gRNA/Cas9-injected embryos and uninjected embryos at stage 20 were pooled and genomic DNA were extracted by using GeneJET Genomic DNA purification Kit (Thermoscientific Co.). Potential off-target sites were amplified by PCR with specific primers (electronic supplementary material, table S3) and T7E1 assay was performed as described [25].

### 2.5. Embryo dissection and Nieuwkoop assays

Animal caps, equatorial zones and vegetal masses were dissected at mid-blastula stage on agarose-coated dishes in 1× MMR, and then cultured in oocyte culture medium [24]. For Nieuwkoop assays, animal caps were assembled on top of vegetal masses such that the blastocoel side of the caps faced the blastocoel side of the vegetal masses. After 1 h of co-culture, animal caps were separated from vegetal masses using tungsten needles, cultured until sibling embryos reached mid-gastrula stage (stage 11) and frozen in batches of 10. The experiment was repeated to ensure the reproducibility of the results described. For the production of anterior/posterior halves (stage 17), embryos were placed in a 2% agarose-coated dish containing 1× MMR, and the vitelline membrane was manually removed using fine forceps; embryos were then transversally bisected using tungsten needles, and equally sized anterior and posterior halves were frozen in batches of four (two embryo equivalent). Left/right halves (stage 22) were produced by the same method, except that embryos were bisected along the midline.

### 2.6. RNA isolation and analysis of gene expression by real-time PCR

Total RNA was extracted and gene expression analysis by RT-PCR was performed as described previously [19], using two whole embryos, three equatorial zones, 10 animal caps, four left/right halves or four anterior/posterior explants. Relative expression values were calculated by comparison with a standard curve generated by 100%, 50% and 10% dilutions of uninjected control cDNA. All samples were normalized to levels of *ornithine decarboxylase* (*odc*), which was used as a loading control. Samples of water alone or controls lacking reverse transcriptase in the cDNA synthesis reaction failed to give specific products in all cases. Standard errors of the mean were not applied to the bar charts of mRNA expression, because they are relative values, not absolute measurements. The method is regarded as ‘semi-quantitative’ for this reason. Experiments were repeated at least three times on different embryo batches to ensure that the pattern of gene expression described was reproducible from one experiment to the next. For splice blocking analysis by gel-based PCR, cDNA was synthesized with random hexamer primers.

### 2.7. Western blot analysis

Western blots were carried out under reducing conditions as previously described [26]. Lysate from five equatorial explants or from 1/5th of five embryos was loaded per lane. Antibodies used were anti-phospho-Smad2 (Cell Signaling Technology; 1 : 500), anti-dp-Erk (Sigma M8159; 1 : 5000) and anti-hemagglutinin (HA) high affinity rat monoclonal antibody 3F10 (Roche 1-867-423; 1 : 2000); anti-α-tubulin (DM1A Neomarkers, 1 : 5000) was used as the loading control. Phopho-Smad2 and dp-Erk western blots were repeated to ensure the reproducibility of the results.

### 2.8. Whole-mount *in situ* hybridization and histology

Prior to *in situ* hybridization, whole embryos were fixed in MEMFA for 2 h and stored at −20°C in 100% ethanol. For bisected gastrulae, whole embryos were fixed in MEMFA for 1 h, then transferred to 1× PBS in agarose-coated dishes in which they were hemisected along the dorsal–ventral axis. Embryo halves were then fixed for an additional hour in MEMFA. *In situ* hybridization was performed as previously described [27].

## 3. Results

### 3.1. Xnr5 mRNA splicing is reduced by a splice blocking morpholino

**Figure 1.**
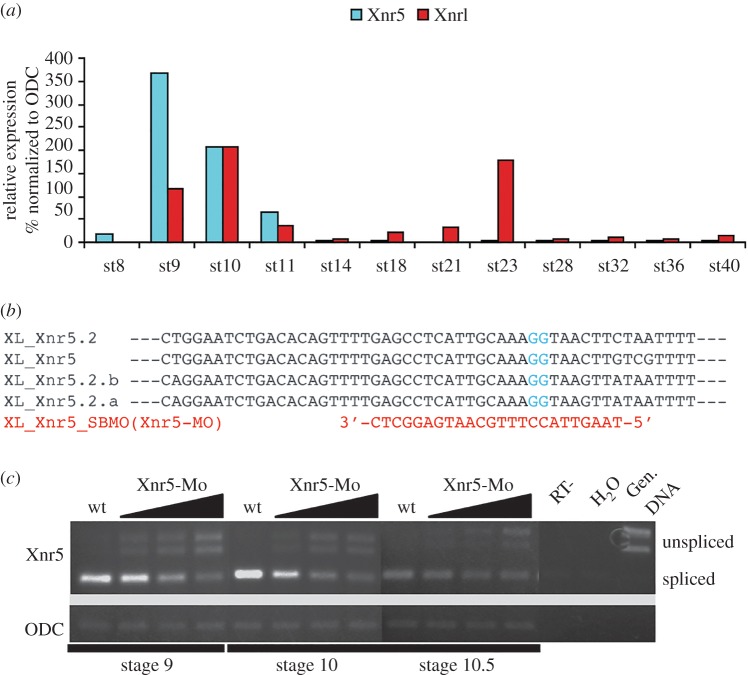
*xnr5* mRNA splicing is reduced by a splice blocking morpholino. (*a*) Real-time PCR (RT-PCR) analysis of the temporal expression of *xnr5* and *xnr1* mRNA in sibling embryos at the developmental stages shown. Both *xnr5* and *xnr1* are expressed at late blastula to mid-gastrula stage. *xnr1* mRNA (but not *xnr5*) is also expressed at late neurula to early tailbud. (*b*) The *xnr5* splice donor MO (Xnr5-MO) targets a conserved exon/intron boundary in all four copies of the *xnr5* gene. (*c*) Gel-based PCR using random hexamer primed cDNAs. Vegetal injection of increasing doses (27, 53 and 107 ng) of the MO reduced splicing of *Xnr5* RNA in a dose-responsive fashion, as testified by the progressive appearance of additional PCR products (unspliced) in Xnr5-MO samples. The appearance of the unspliced product correlates with decreases in the mature (spliced) product. *odc* is used as RNA loading control.

Xnr5 has previously been shown to be expressed in the vegetal mass of blastula stage embryos, and particularly enriched on the dorsal side. To confirm its temporal expression pattern, we compared *xnr5* and *xnr1* mRNA levels of expression over a temporal series of development by RT-PCR. Figure 1*a* shows that Xnr5 is expressed only during a 6 h period from the blastula to the early gastrula stage, with no detectable expression after this. By contrast, the related Nodal gene, *xnr1* has subsequent waves of expression during the neurula and early tailbud stages (figure 1*a*) [4,28].

The Xnr5 gene is amplified in the *Xenopus* genome, with transcripts known to be derived from three copies of the gene [7]. Recently finished genome assembly shows that there are four copies of the gene in both *X. laevis* and *X. tropicalis* (www.xenbase.org). To develop a loss of function strategy, a splice blocking antisense MO was designed (figure 1*b*) because there is no antibody available for Xnr5, and published translational blocking MOs have so far only been tested for their ability to block the activity of overexpressed *xnr5* mRNA [17]. This MO targets a conserved exon/intron boundary in all four copies of the *xnr5* gene, although a high dose (60 ng) was required for efficient reduction of the spliced forms of *xnr5* (figure 1*c*). Because of the high dose of oligo used, particular attention was paid to the ability of *xnr5* mRNA to rescue the observed phenotypes, to confirm the specificity of the effects.

### 3.2. Xnr5 acts as a mesoderm inducer and a negative regulator of ectodermal gene expression

**Figure 2.**
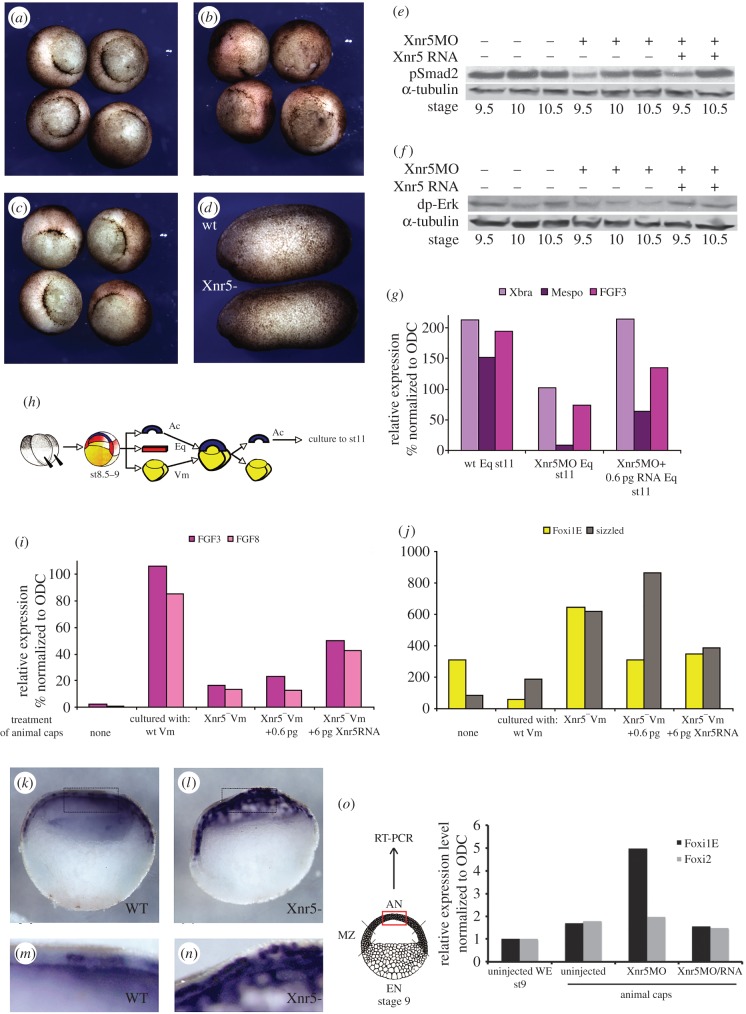
Xnr5 is a mesoderm induction and ectoderm repression signal. (*a*) Control, (*b*) Xnr5-depleted and (*c*) Xnr5-depleted embryos injected with 0.6pg of *xnr5* RNA. Xnr5-depleted embryos (*b*) usually develop with a slight gastrulation delay (depicted by the delay in blastopore closure: (*b*) compared with (*a*)) which is rescued by reintroduction of *xnr5* RNA (20/20 cases). (*d*) Wild-type (upper) and Xnr5-depleted (lower) embryos at early tailbud stage. No morphological alteration of Xnr5-depleted embryos compared with controls is noticed through this stage. (*e*) Western blot analysis showing phosphorylated Smad2 levels in control and Xnr5-depleted gastrulae (Xnr5MO), and embryos injected with both MO (Xnr5MO) and 0.6pg *xnr5* mRNA. (*f*) Western blot analysis showing phosphorylated Erk in equatorial explants of control, Xnr5-depleted and Xnr5-depleted embryos injected with *xnr5* mRNA at the four-cell stage, at early gastrula stages. (*g*) RT-PCR analysis of the expression of mesodermal markers, *xbra*, *mespo* and *fgf3* mRNAs, in equatorial explants from control (wt Eq), Xnr5-depleted (Xnr5MO Eq) and Xnr5-depleted embryos injected with *xnr5* mRNA at the four-cell stage (Xnr5MO + 0.6 pgRNA). (*h*) Design of the Nieuwkoop assay used to produce the animal caps (Ac) analysed by RT-PCR in (*i*) and (*j*). (*i*) Mesodermal markers, *fgf3* and *fgf8* are induced in wild-type animal caps by wild-type vegetal masses (wt Vm), reduced with Xnr5-depleted vegetal masses (Xnr5^−^Vm), and rescued by co-culture with Xnr5-depleted vegetal masses injected with 6pg *xnr5* mRNA (Xnr5^−^Vm + 6 pg); (*j*) ectodermal markers (*foxi1E* and *sizzled*) are upregulated as a result of Xnr5 depletion. (*k*–*n*) Whole mount *in situ* hybridization of *foxi1E* mRNA in (*k*,*m*) bisected control and (*l*,*n*) Xnr5-depleted embryos. Panels (*m*) and (*n*) represent the magnification of boxed areas in (*k*) and (*l*), respectively. (*o*) RT-PCR analysis of the expression of ectodermal genes, *foxi1E* and maternal *foxi2* in small animal cap explants from control (uninjected), Xnr5-depleted (Xnr5MO) and Xnr5-depleted embryos injected with 0.6 pg of *xnr5* mRNA at the four-cell stage (Xnr5MO/RNA). The diagram shows the area (red box) of the animal cap explants for the experiment. AN, animal cells; MZ, marginal zone; EN, endodermal cells.

To examine the roles of Xnr5, MO was injected into all four vegetal cells at the eight-cell stage, and embryos allowed to develop. Control and Xnr5-depleted embryos developed normally to the gastrula stage, and formed blastopores, although the blastopores were variably (0–60 min) delayed and less smooth than those of controls or of Xnr5-depleted embryos in which a small dose of *xnr5* mRNA was injected at the four-cell stage (figure 2*a*–*c*).

To test whether Xnr5 plays a role in TGF-β signalling before and during gastrulation, wild-type controls, Xnr5-depleted embryos and Xnr5-depleted embryos injected with 0.6pg *xnr5* mRNA were frozen at late blastula through mid-gastrula stages (2 h intervals) and analysed for phospho-Smad2 levels by western blotting. Smad2 phosphorylation was reduced compared with controls at the late blastula stage (in three experiments) but returned to normal levels during gastrulation (figure 2*e*), consistent with delayed blastopore formation. The reintroduction of *xnr5* mRNA enhanced Smad2 phosphorylation, but not until the gastrula stage, suggesting that the posttranslational modification of Xnr5 or of Smad2 phosphorylation downstream of it does not mirror the endogenous protein's activity; however because the rescue worked for dp-ERK (figure 2*f*), this could also be a matter of differential sensitivity of the respective pathways to the exogenous Xnr5. Xnr5 and Vg1 activate an activin-like signalling pathway, and both have been shown to induce FGF8 [24,29]. Moreover, it has been shown that a functional FGF signalling pathway is required for the induction of some mesodermal genes downstream of activin [29,30]. Indeed, the earliest activation of dp-ERK is seen in the dorsal marginal zone [31], and among the earliest genes activated in mesoderm, Fgf4 and Fgf8 are most probably induced by nodal signalling [32]. As nodal signalling downstream of VegT is known to be required for mesoderm induction, we next tested Xnr5's specific role in three ways. First, we dissected equatorial explants (mesodermal precursors) from control, Xnr5-depleted embryos and Xnr5-depleted + *xnr5* mRNA injected late blastulae, early and mid-gastrulae, and compared phospho-Erk levels. Figure 2*f* shows that Xnr5 depletion caused a reduction in activation of Erk that was rescued by the re-introduction of *xnr5* mRNA (0.6 pg). Second, we analysed by RT-PCR the expression of molecular markers of mesoderm formation in sibling explants frozen at the mid-gastrula stage. Xnr5 depletion caused a reproducible reduction in the expression of mesodermal genes including *xbra* (the same as *t*), *mespo* (the same as *msgn1* [33]) and *fgf3* [34], which was partially rescued by re-introduction of *xnr5* mRNA (figure 2*g*).

Third, Nieuwkoop assays were performed (figure 2*h*; electronic supplementary material, figure S4), in which wild-type mid-blastula animal caps were co-cultured with wild-type, Xnr5-depleted, or depleted + *xnr5* mRNA injected vegetal masses for 1 h during the late blastula stage (figure 2*i*,*j*). This showed that mesodermal genes including *fgf3* and *8* were induced in animal caps that were co-cultured with wild-type vegetal masses, and this induction was reduced when vegetal masses were depleted of Xnr5 and partially rescued by *xnr5* mRNA, injected into the Xnr5-depleted embryos at the four-cell stage. By contrast, the ectodermal gene *foxi1E* and the animally and ventrally expressed *sizzled* mRNA were upregulated in this assay (figure 2*i,j*) [35,36]. The upregulation of *foxi1E* was confirmed by *in situ* hybridization analysis of wild-type and Xnr5-depleted hemisected embryos at the early gastrula stage. Figure 2*k*–*n* shows that, in wild-type embryos, *foxi1E* mRNA is expressed in a salt and pepper fashion throughout the animal hemisphere, and its level of expression is increased as a result of Xnr5 loss of function. In order to confirm that Xnr5 regulates the expression of *foxi1E* in the animal cap*,* we dissected relatively small pieces of animal regions from uninjected, Xnr5-depleted and rescued whole embryos. The RT-PCR data show the upregulation of *foxi1E* in Xnr5-depleted animal tissue can be rescued by injecting 0.6 pg of *xnr5* mRNA (figure 2*o*). However, Xnr5 depletion did not cause ectopic activation of *foxi1E* expression. We conclude from this that Xnr5 is required to activate mesodermal zygotic gene expression in equatorial cells and to downregulate the level of ectodermal gene expression in animal cells at the early gastrula stage.

### 3.3. Xnr5 and Vg1 share roles in mesoderm induction

Despite these changes in target gene expression, sibling Xnr5-depleted embryos developed relatively normally through the gastrula, neurula and tailbud stages (figure 2*d*), suggesting that its function may be redundant with another TGF-β-signalling molecule/s. As well as *xnr5*, the related nodal *xnr6* is expressed at the mid-blastula transition and is enriched in a similar area to *xnr5*, and is thus a candidate for sharing *xnr5*'s function [8]. However, in preliminary experiments, Xnr6 depletion alone (using a specific morpholino) did not impair mesoderm induction, and its combined depletion with Xnr5 did not cause enhanced defects in mesoderm induction (data not shown).

**Figure 3.**
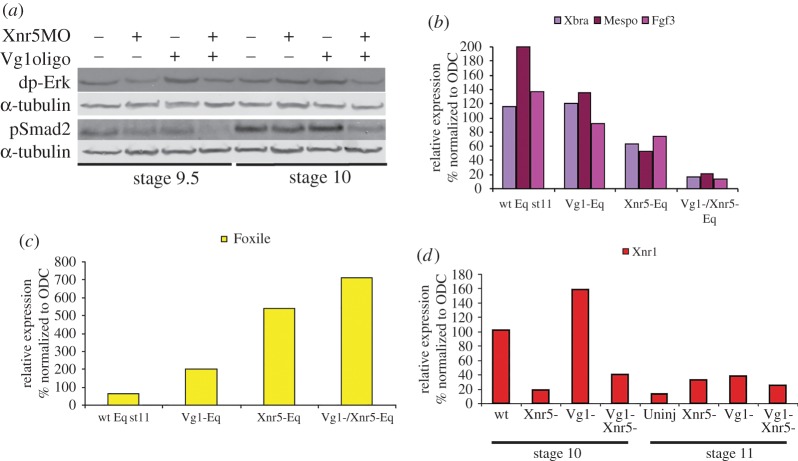
Xnr5 and Vg1 share roles in mesoderm induction and Foxi1E inhibition. (*a*) Western blot analysis of control, Xnr5^−^, Vg1^−^ and Vg1^−^/Xnr5^−^ equatorial explants at the early gastrula stage for dp-Erk and pSmad2; double depletion of both Xnr5 and Vg1 (lanes 4 and 8) causes a more severe reduction in dp-Erk and pSmad2 than single depletion. (*b*,*c*) RT-PCR analysis of the expression of mesodermal markers *xbra*, *mespo* and *fgf3* (*b*) and ectodermal marker *foxi1E* (*c*) in such explants; double depletion (Xnr5^−^/Vg1^−^) has a more severe effect on both mesodermal (decrease) and ectodermal (increase) markers. (*d*) Xnr5 depletion and Vg1 depletion have opposite effects on the expression of *xnr1* mRNA at the early (stage 10) and mid-gastrula stage (stage 11).

Vg1 is the first activin-type TGF-β expressed in the early embryo, as it is inherited from the oocyte as a maternally stored mRNA and protein [37,38], and is enriched dorsally, like Xnr5 [19,24]. We therefore tested whether Vg1 cooperates with Xnr5 in mesoderm induction, by reducing *xnr5* and *vg1* mRNA alone or together from the early embryo using antisense-mediated depletion, as described previously [39]. Here, low doses of Xnr5 morpholino and Vg1 phosphorothioate–phosphodiester oligos were injected into oocytes before fertilization, so that each alone did not cause an extreme phenotype. Figure 3*a* shows that the double depletion of both *vg1* and *xnr5* mRNA caused severe reduction in Erk and Smad2 phosphorylation (lanes 4 and 8) compared with Xnr5 or Vg1 depletion alone. This effect on mesoderm induction was confirmed by the analysis of mesoderm markers in equatorial explants from sibling embryos, where the double depletion severely reduced the expression of *xbra*, *mespo* and *fgf3* (figure 3*b*). Similar to Xnr5, Vg1 depletion also caused the upregulation of the ectodermal gene *foxi1E* (figure 3*c*) [35]. These data show that Xnr5 acts additively with Vg1 in mesoderm induction, suggesting that the endogenous expression of Vg1 in Xnr5-depleted embryos might be sufficient to sustain mesoderm induction when Xnr5 is depleted, and allow embryos to develop through the gastrula stage. However, Vg1's role is not identical to that of Xnr5, as the loss of Xnr5 function caused a reduction in the expression of the related nodal gene, *xnr1*, while Vg1 depletion caused an upregulation of *xnr1* expression (figure 3*d*) [24].

### 3.4. Xnr5 is required for the later expression of *Xnr1* mRNA

**Figure 4.**
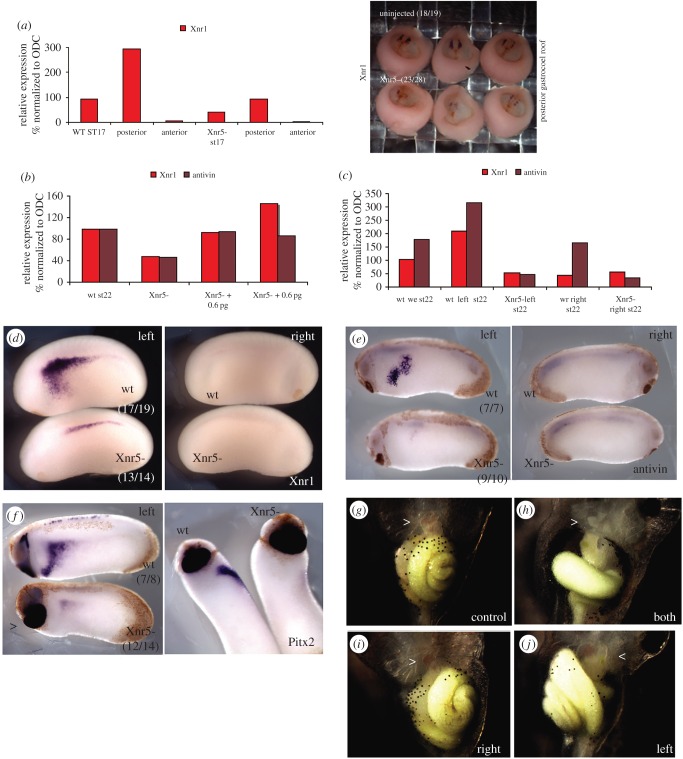
Xnr5 is required for the later expression of *xnr1* mRNA. (*a*) RT-PCR (left panel) and *in situ* hybridization (right panel) analyses of *xnr1* mRNA in anterior/posterior bisected embryos; *xnr1* mRNA is only detected in posterior explants of controls embryos at neurula stage (stage 17), and this strong expression of *xnr1* is severely reduced in Xnr5-depleted (Xnr5^−^) explants. (*b*) RT-PCR analysis of the expression of *xnr1* and *antivin* mRNA in whole embryos at the early tailbud stage (stage 22); their expression is reduced in Xnr5-depleted embryos and partially rescued by reintroduction of 0.6 and 6 pg of *xnr5* mRNA. (*c*) RT-PCR analysis of whole embryos (we) or left or right half embryos showing the enrichment of *xnr1* and *antivin* mRNA on the left side of controls (wt left) at early tailbud stage (stage 22); the high levels of expression are lost in Xnr5-depleted embryos (Xnr5^−^ left). (*d*–*f*) *In situ* hybridization analysis of *xnr1* (*d*), *antivin* (*e*) and *pitx2* (*f*) mRNA expressions at tailbud stage. *xnr1* as well as *antivin* and *pitx2* transcripts are detected in the left (but not right) lateral plate mesoderm in wild-type embryos (wt); the left-sided expression of these transcripts is lost in Xnr5-depleted embryos (Xnr5-); note that the expression of *pitx2* in the cement gland (arrow) is not reduced. (*g*–*j*) Wild-type (*g*) or tadpole injected Xnr5MO in all four cells (*h*) in two right blastomeres (*i*) or two left blastomeres (*j*) at four-cell stage; injection of Xnr5MO in all 4 cells ((*h*) compared with (*g*)) causes an incomplete gut looping, often with a small and midline linear heart (arrow-head in (*g*–*j*)); injecting the same total dose of MO into the two right cells (*i*) causes no obvious effect, while injecting into the two left cells (*j*) causes an inversion of gut and heart looping (note the position of the gall-bladder, just below the arrow-head, is inverted in (*j*) compared with (*g*)).

Unlike *xnr5*, *xnr1* is expressed in the posterior gastrocoel roof at the neurula stage [28,40]. Therefore, we asked whether this later *xnr1* expression was affected in Xnr5-depleted embryos. In stage-matched control and Xnr5-depleted embryos, *xnr1* expression in neurula stage embryos was reproducibly reduced by Xnr5 depletion both in whole embryos and in posterior half embryos compared with controls by both RT-PCR (figure 4*a*, left panel) and *in situ* hybridization (figure 4*a*, right panel).

The first evidence of left/right asymmetry at the mRNA level is marked by the third wave of Xnr1 expression, which occurs in the left LPM of the early tailbud stage embryo [12,14,41]. We tested whether Xnr5 depletion affected tailbud expression of *xnr1* by real-time RT-PCR analysis and by *in situ* hybridization. Figure 4*b* shows that *xnr1* levels were reduced in Xnr5-depleted tailbud stage embryos and the level was rescued by 0.6 pg of *xnr5* mRNA injected at the four-cell stage. 6 pg of *xnr5* mRNA caused an overexpression of *xnr1* (figure 4*b*). Figure 4*c*,*d* shows the reduction in expression of left lateral *xnr1* mRNA in Xnr5-depleted embryos compared with controls. This effect was highly reproducible (in three experiments), and was not simply due to a delay in expression, because *in situ* hybridization for *xnr1* at later tailbud stages did not show a late appearance of left-sided *xnr1* mRNA in Xnr5-depleted embryos (data not shown).

To confirm the role of Xnr5 in regulating laterality, we next examined the expression pattern of the nodal inhibitor *antivin*, and the transcription factor *pitx2*, both of which are known to be direct targets of left sided Xnr1 signalling activity [42,43]. Both *antivin* and *pitx2* expression in the left LPM were severely reduced in Xnr5-depleted embryos (figure 4*e,f*), but *pitx2* expressed in the cement gland area was not affected (arrow-head in figure 4*f*).

As Xnr1 has been shown to be required for left/right axis formation and normal heart and gut orientation [28,40], and we show here that *xnr1* is downstream of Xnr5, we examined whether asymmetric Xnr5 depletion affected heart and gut positioning. Xnr5 MO was injected into either all four cells of the four-cell stage embryo, or into the two left or two right blastomeres. The site of injection was confirmed at the neurula stage by the presence of fluorescein-tagged MO on the left or right side. When MO was injected on both sides, the embryos developed with small midline hearts (arrow-head) and abnormally rotated guts (figure 4*h*). The same dose of MO restricted to the right side caused the majority of embryos to develop with normal laterality (figure 4*i*), while on the left side it caused an average of 46% in four experiments to develop with heterotaxia or situs inversus (figure 4*j*; electronic supplementary material, table S1). We conclude that Xnr5 regulates the laterality of the early embryo by regulating the expression of the known laterality inducer Xnr1.

### 3.5. Xnr5 depletion causes heart dysmorphogenesis

**Figure 5.**
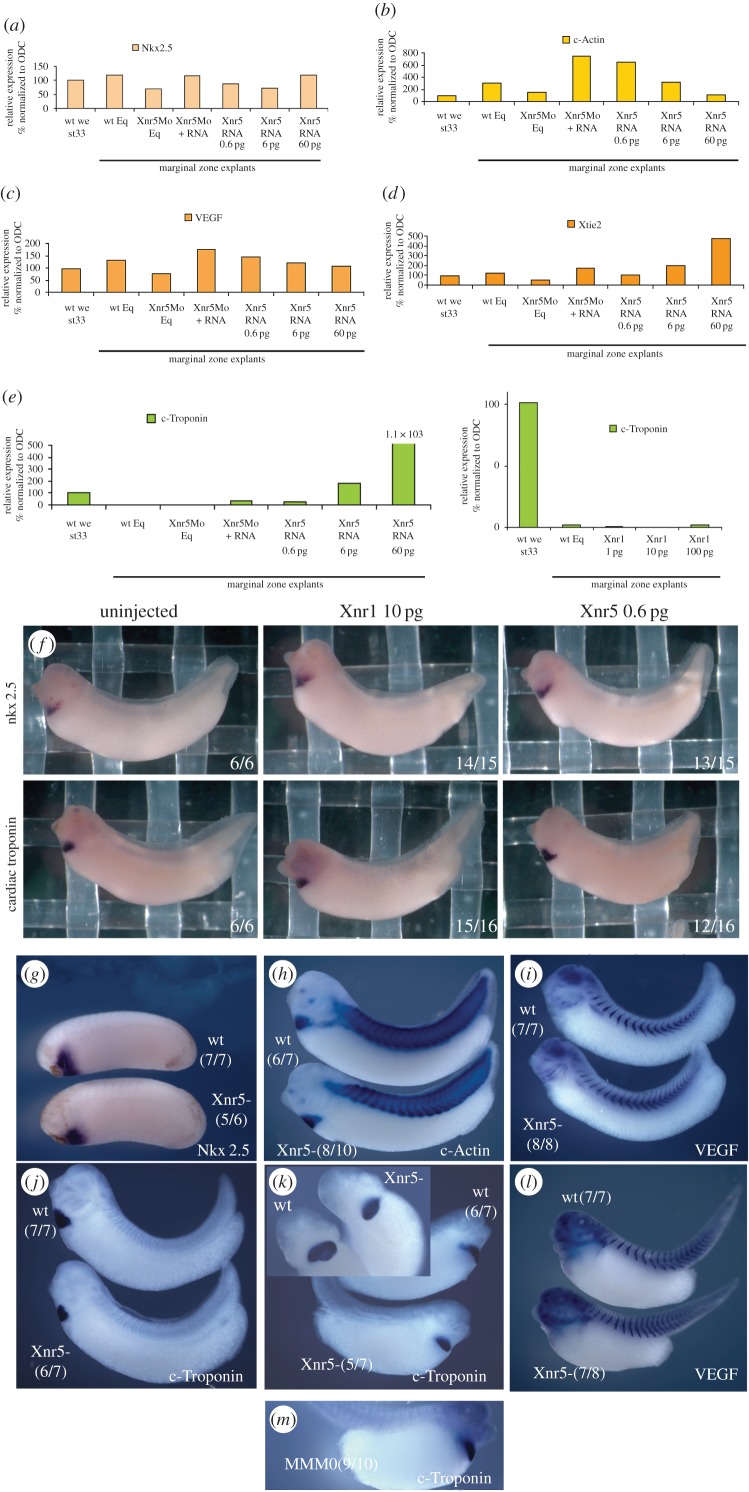
Xnr5 depletion causes heart dysmorphogenesis. (*a*–*e*) RT-PCR analysis of the expression of cardiac markers *nkx2.5* (*a*), *c-actin* (*b*) and *c-troponin* (*e*) and vascular markers *vegf* (*c*) and *tie2* (*d*) in wild-type (wt Eq) Xnr5-depleted (Xnr5Mo) Xnr5-depleted injected with 0.6 pg of *xnr5* mRNA (Xnr5Mo + RNA) and equatorial explants injected with increasing doses of *xnr5* mRNA (*xnr5* RNA 0.6 pg, 6 pg and 60 pg), dissected at late blastula stage and cultured to the tailbud stage (stage 33). Xnr5 depletion causes a reduction of the expression of *nkx2.5*, *c-actin*, *vegf* and *tie2* whose levels are re-established by the reintroduction of *xnr5* mRNA; overexpression of *xnr5* has a mild induction effect only on *tie2* expression. *cardiac troponin* (*e*) is not expressed in wild-type explants (wt Eq) but is dose-dependently induced by *xnr5* mRNA. RT-PCR analysis showing that overexpressing *xnr1* mRNA (1 pg, 10 pg and 100 pg) does not induce *c-troponin* in equatorial explants. (*f*) Whole mount *in situ* hybridization of 10 pg of *xnr1* mRNA injected embryos and 0.6 pg of *xnr5* mRNA injected embryos for *nkx2.5* and *cardiac troponin* (*tnni3*). (*g*–*m*) *In situ* hybridization analysis of *nkx2.5* (*g*), *c-actin* (*h*), *vegf* (*i*,*l*) and *c-troponin* (*j*,*k*,*m*) in control (wt), Xnr5-depleted (Xnr5-) or embryos injected with a mismatched version of Xnr5Mo (MMMO) containing 5 base exchange. The expression of early heart field markers *nkx2.5* (*g*, stage 26) and *c-actin* (*h*, stage 33) is unchanged in Xnr5-depleted embryos when compared with controls. The same observation is true for the expression of *vegf* at stage 32 (*i*) and stage 41 (*l*). The expression domain of *c-troponin* is reduced in Xnr5-depleted embryos at stage 33 (*j*) and stage 41 (*k*); a mismatched version of Xnr5MO has no effect on the expression of *c-troponin* ((*m*) compared with wt in (*k*)).

As Xnr5 depletion caused early defects in mesoderm formation and later abnormalities of LPM and heart development, we analysed the effects of Xnr5 depletion, rescue and overexpression on equatorial (mesodermal precursor) explants dissected at the late blastula stage and cultured until sibling embryos reached tailbud stages for the expression of a panel of heart and blood vascular system markers by RT-PCR. Using tissue explant to study cardiogenesis has been performed on both *Xenopus* and chicken [44–47]. We also included markers of lymphatic development and mesonephros formation (summarized in electronic supplementary material, table S2). *In situ* hybridization experiments were used to confirm the findings in whole embryos. The markers of the early heart field, including *nkx2*.5 (the same as *nkx2-5* or *NK2 homeobox 5*; figure 5*a*) and *cardiac actin* (the same as *actc1*; figure 5*b*) were slightly reduced in their expression in MO-explants (Xnr5 Mo Eq) in early tailbud, and this was rescued by Xnr5 expression (Xnr5Mo + RNA). *In situ* hybridization data on whole embryos showed that the heart field of Xnr5-depleted embryos was specified but smaller than in controls, as evidenced by levels and position of *nkx2*.5 (figure 5*g*) and *cardiac actin* (figure 5*h*). Although vascular markers *vegf* (figure 5*c*,*i*) and *tie2* (the same as *tek*; figure 5*d*) were also slightly reduced at stage 33, their expression pattern was normal at the late tailbud stage (figure 5*l*), suggesting that vascular specification was not impaired.

Unlike the other vascular and cardiac markers, *cardiac troponin* was not expressed in wild-type marginal zone explants, dissected at the late blastula stage and cultured to the tailbud stage, presumably because its expression requires an endodermal signal. Moreover, when we cultured the explants until the sibling control embryos reached stage 33, we did not see any beating heart in explants. This suggests that our experimental conditions were not sufficient to induce a full cardiogenic programme in explants. Recently, it has been shown that anterior endoderm from stage 10.25–10.5 can induce late cardiac markers in conjugated animal cap tissue [45].

When xnr5 mRNA was overexpressed in such marginal zone explants, it caused a dose-responsive upregulation of cardiac troponin expression (figure 5*e*). Although in whole embryo the overexpression of xnr5 mRNA could be applied only with 0.6 pg mRNA in order to avoid early gastrulation defects, this low dose still caused weak upregulation of cardiac genes (figure 5*f*, right panel). Conversely, the amount of cardiac troponin expression was reduced in Xnr5-depleted embryos by *in situ* hybridization analysis at both the early (figure 5*j*) and late tailbud stage (figure 5*k*).

As *cardiac troponin* expression is sensitive to *xnr5* expression, and *cardiac troponin* is known to be important for sarcomere formation and cardiac contractility [48–50], it may be an important factor in the heart dysmorphogenesis resulting from Xnr5 loss of function.

Xnr5 regulation of *cardiac troponin* could be dependent or independent of its regulation of left lateral *xnr1* expression. Previous studies have suggested that Xnr1 loss of function affected only left/right patterning of the viscera and no other aspects of embryonic patterning [28]. To confirm this, we asked whether *xnr1* also regulates *cardiac troponin* by examining whether overexpressing *xnr1* mRNA in equatorial explants affects *cardiac troponin* expression. Although we could overexpress 60–100 pg of Xnr mRNAs to check whether they could induce cardiogenic markers in explants, for whole-embryo analyses at later stages, we had to limit the amount of Xnr5 mRNA to a very low dose in order to avoid early gastrulation defects. It has been shown that even 0.6 pg of Xnr5 mRNA is sufficient to induce mesodermal marker *Xbra* and cause both head reduction and abnormal protrusions when the mRNA is injected into one ventral cell of four-cell stage embryos [19]. Figure 5*e,f* shows that *xnr1* overexpression in explants or whole embryo did not induce *cardiac troponin*, unlike Xnr5, suggesting that either Xnr5 regulates *xnr1* and *cardiac troponin* by different pathways or Xnr5 and Xnr1 are differentially potent in their inductive activities.

At the swimming tadpole stage (stage 45–46) a large majority of Xnr5-depleted embryos developed severe oedema. Oedema could be the result of the failure of cardiovascular development, but it could also result from abnormal development of the urinary system or lymphatic system, or be due to a non-specific effect due to morpholino injection. While we discounted failures in pronephros and lymphatic vessel formation (*prox1*) (data not shown), non-specific morpholino effects could not be discounted in these experiments, because a mismatch morpholino, which did not cause any effects on the earlier phenotypes documented above, did cause oedema (data not shown). We conclude that oedema could be a non-specific result of morpholino injection.

### 3.6. Targeted genomic disruption of the *Xnr5* genes impairs the expression of *Xnr1* in the left lateral plate mesoderm

The knockdown phenotype of Xnr5 SBMO injected embryo is not the same as for embryos injected with either cleavage mutant form of *xnr5* mRNA or endoplasmic reticulum (ER)-retaining *xnr5-KDEL* tagged mRNA [51,52]. The SBMO used in this study targets Xnr5 pre-mRNA and interferes with its splicing event towards mature mRNA. A cleavage mutant is supposed to bind to endogenous/wild-type Xnr5, but it has been reported that cm-Xnr5 can also inhibit Xnr6's activity upon overexpression assay. Xnr5-KDEL used a similar idea, but in ER, the ER retention signal (KDEL) keeps Xnr5 pro-protein in ER, and in using this tagged Xnr5 mRNA one has to assume that the resulting tagged protein is able to capture and sequester the endogenous/wild-type Xnr5 in the ER. Recent implantation of CRISPR/Cas-mediated genome editing technique to *Xenopus* species allows us to test which phenotype is derived from the actual disruption of the function of the endogenous Xnr5 [53–56].

**Figure 6.**
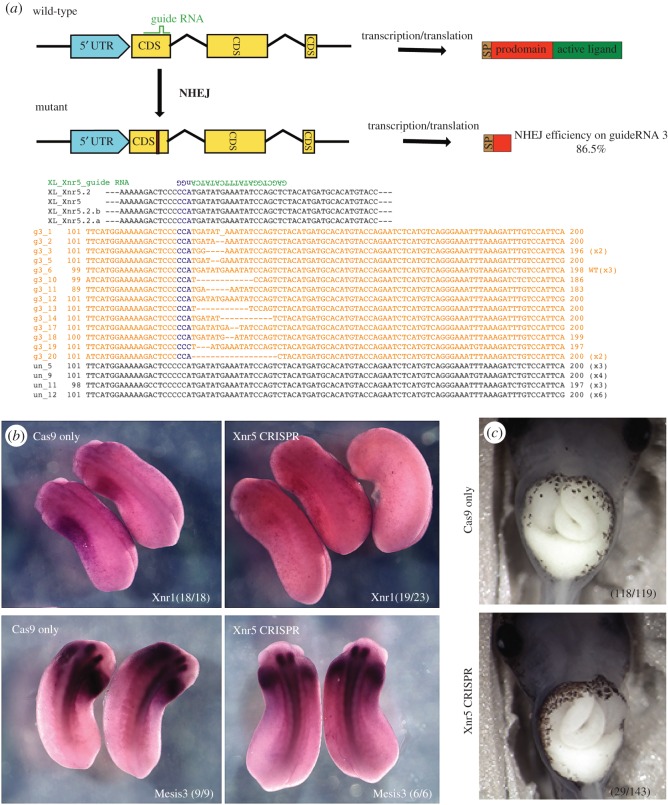
CRISPR/Cas9 mediated Xnr5 knockout embryos lost asymmetric *xnr1* expression. (*a*) Schematic description of CRISRP/Cas9 mediated NHEJ event on *xnr5* genes and clonal sequencing analysis of *xnr5* mRNA at stage 9 embryos. (*b*) *In situ* hybridization analysis of x*nr1*, *meis3* mRNA expression at tailbud stage. *xnr1* transcripts are detected in the left (but not right) lateral plate mesoderm in Cas9-injected embryos (Cas9 only); the left-sided expression of these transcripts is lost in Xnr5 gRNA/Cas9-injected embryos (Xnr5 CRISPR); note that the expression of *meis3* is not altered. (*c*) Xnr5 CRISPR mutants developed heterotaxia (including situs inversus).

To make a Xnr5-targeting gRNA, we scanned all Xnr5 coding sequences for potential sites of the form GGN_20_NGG using the ZiFiT targeter webtool [57,58] and found several candidate sites that conversed among all Xnr5 genes. These sites lie in the 5′ end of the ORFs within the pro-protein domain and are conserved in all four genomic copies of Xnr5 (figure 6*a*). Upon double-strand break/NHEJ event, we expected to have non-sense mutation of *xnr5* genes throughout the embryo, due to insertion/deletion events. Two Xnr5 guide RNAs (Xnr5_g1 and Xnr5_g3; target sites and potential off-target sites are listed in the electronic supplementary material, table S3) were selected and analysed by T7E1 assay.

A mixture of 600 pg NLS-SpCas9 protein and 300 pg of each Xnr5 gRNA was injected into one-cell-stage *X. laevis* embryos and they were cultured at 23°C until they reached late tailbud stages. We fixed embryos and performed whole-mount *in situ* hybridization with *xnr1* to check the laterality and *meis3* to see the symmetrical development of hindbrain/LPM. Both gRNAs cause a downregulation of *xnr1* and *pitx2* on the left side of the injected embryos consistent, with the data from Xnr5 SBMO injected embryos (figure 6*b* for Xnr1; electronic supplementary material, figure S3). The expression pattern of *meis3* has not been altered by Cas9/gRNA components. Similar to Xnr5 SBMO morphants, Xnr5 CRISPR mutants developed laterality defect at the tadpole stage. At later stage, Xnr5 CRISPR mutants developed heterotaxia (including situs inversus) (13–26%) like Xnr5 SBMO morphants, but oedema was observed at a far lower frequency (8–12%). Unlike Xnr5 SBMO morphants, Xnr5 CRIPSR mutants exhibit a low frequency (5–8%) of craniofacial malformations (triangle-shaped head with partial fused eyes) (figure 6*c*; electronic supplementary material, figure S3 and table S4).

As there are 16 Xnr5 transcripts in *X. laevis* and we do not have a tool to clarify which ones are preferentially expressed, we isolated mRNA from late blastula (stage 8.5/9) embryos of either Xnr5 gRNA injected or uninjected sibling control. The clonal sequencing analysis of mRNA revealed that 85% (17/20) of the selected clones encoded transcripts whose translation would produce a truncated Xnr5 mutant protein and only 15% (3/20) of the mRNAs would produce the normal Xnr5 pro-protein. This level is comparable to the remaining fully matured *xnr5* mRNA detected upon injection of 60 ng of SBMO (figure 1).

To address whether Xnr5_g1 and g3 might have off-target activity, we chose top 11 potential off-target sites and performed T7E1 assay with injected embryos compared with uninjected siblings. Consistent with previously reported data from other *Xenopus* research groups [53,56], we did not observe any T7E1-measurable off-target effects of the gRNA (electronic supplementary material, figure S1).

## 4. Discussion

### 4.1. Specific and non-specific effects of morpholino oligos

In the experiments reported here we show that Xnr5 has roles at three time points in early development. First, at the gastrula stage it is required together with Vg1 for mesodermal gene expression and repression of the ectodermal gene *foxi1E*. Confidence in the specificity of the phenotype is based on the fact that we can partially rescue the effects by injecting *xnr5* mRNA, and that a mismatch oligo does not cause these effects. Second, at the early tailbud stage it is required for the left-sided expression of *xnr1*, *antivin* and *pitx2*, and we show that the expression of *xnr1* and *antivin* is partially rescued by injection of *xnr5* mRNA.

Third, at the tailbud stage, Xnr5 is required for the correct size of the heart field, as evidenced by the expression of *cardiac troponin* mRNA. Although this could be the result of an early defect in mesoderm induction, the evidence that this effect is specific rests upon the fact that *cardiac troponin* expression level is reduced in Xnr5-depleted embryos, that overexpression of Xnr5 causes a dose-responsive increase in *cardiac troponin* expression in explants and an expansion of the expression domains of *nkx2*.5 and *cardiac troponin* in the whole embryo at the tailbud stage (figure 5*f*). Furthermore, a mismatch oligo does not cause this effect (figure 5*m*). This latter phenotype is not confirmed by a rescue experiment, as Xnr5-depleted embryos injected with *Xnr5* mRNA do not develop normally to the tailbud stage. We have previously shown that the embryo is extremely sensitive even to very low doses of *xnr5* mRNA (0.6 pg [19]), and the inability to rescue is probably caused by the incorrect spatio-temporal location and amount of introduced Xnr5 protein.

After extensive analysis, we believe that oedema at the swimming tadpole stage in Xnr5-depleted embryos is likely to be a non-specific morpholino effect, because a mismatch morpholino, which we confirmed did not block splicing, caused none of the early phenotypes, but did cause oedema at the swimming tadpole stage, when injected in 60 ng doses. Why high doses of morpholino oligos should cause effects so late in development is not clear. Studies in zebrafish suggest that ‘off-target effects’ may include upregulation of a non-functional truncated form of the tumour suppressor, p53 [59], although why this should happen is not understood. This highlights the importance of adequate controls for morpholino experiments. Simple and efficient CRIPSR/Cas-mediated genome editing method recapitulated the phenotype of Xnr5 SBMO. Since the gene has single wave of expression during early embryogenesis, using direct genome editing can be very powerful to prove target gene's function. Our results reinforce the idea that using this tool can be a valuable asset as a proper control for morpholino experiments.

### 4.2. Xnr5 expressed at the blastula stage regulates laterality

The fact that Xnr5 regulates *xnr1* expression at the gastrula stage is not unexpected, because Xnrs have been shown to cross-regulate each other in overexpression experiments, and it is known that *xnr1* is regulated by nodal signalling [18,60,61]. It is surprising that it regulates asymmetric Xnr1 expression 24 h later, at the tailbud stage. Several mechanisms could be responsible. First, Xnr5 may be secreted and signal only at the beginning of gastrulation, and lie at the top of a cascade of gene expression that results in the later asymmetric expression of *xnr1*. Alternatively, Xnr5 may be a stable protein whose signalling is regulated so that it acts both at the gastrula and neurula/tailbud stages to initiate *xnr1* expression at these different times directly. Studies in zebrafish embryos and cells in culture suggest that nodal and nodal-related protein (Cyclops) are not stable and do not signal at long range [62,63], although the related zebrafish protein *squint* does signal over long range, and therefore may be more stable [64,65]. Chick nodal and *Xenopus* Xnr2 have both been reported to diffuse over long distances [66,67], but protein stability has not been measured. In preliminary experiments, we found that overexpressed Xnr5 protein is stable until the neurula stage, and therefore could activate late Xnr1 expression directly (electronic supplementary material, figure S2), but this may not necessarily reflect the behaviour of endogenous protein.

How does Xnr5 expression result in Xnr1 asymmetric expression in the left/right axis of the LPM? We analysed whether *xnr5* mRNA is itself asymmetrically expressed, and found no evidence that *xnr5* mRNA or its regulators, *wnt11*, *foxH1* and *vegt*, were expressed asymmetrically in the left/right axis (data not shown). We cannot rule out, however, that small differences in RNA levels or differences in protein stability may exist on one side versus the other.

Previous studies have shown that at the early neurula stage, monocilia, driving leftward flow in the gastrocoel cavity, determine laterality in *Xenopus* [68]. We examined here whether monocilia were abnormal in Xnr5-depleted embryos, using confocal microscopy to examine the monocilia on the roof of the gastrocoel cavity, and m-cherry tubulin to label cilia in living explants (data not shown). We found no evidence that Xnr5 depletion affected monocilia formation or beating in the posterior gastrocoel roof.

No mechanistic link has yet been made between the other known early regulators of left/right asymmetry in *Xenopus*, including gap and tight junction communication [11,69], serotonin levels [70] and microfilament induced torsion [71], with the later events involved in left/right axis (gastrocoel monocilia, asymmetric *xnr1* expression). Previous studies have suggested that the maternal TGF-β, Vg1, is an essential early regulator of laterality in *Xenopus* [72,73]. We have shown here and elsewhere that both the overexpression and the loss of Vg1 function results in upregulation of *xnr1* expression at the gastrula stage (figure 3*d* [24]), while Xnr5 loss of function downregulates *xnr1* expression. It seems likely that a careful balance of Xnr5 and Vg1 signalling is necessary to establish the correct spatio-temporal expression pattern of Xnr1 activity.

## Data accessibility

The datasets supporting this article have been uploaded as part of the electronic supplementary material.
